# A role of CD20^+^ T cells in early multiple sclerosis

**DOI:** 10.3389/fimmu.2025.1582535

**Published:** 2025-05-19

**Authors:** Marina Rode von Essen, Marie Mathilde Hansen, Sahla El Mahdaoui, Victoria Hyslop Hvalkof, Rikke Holm Hansen, Jørgen E. Nielsen, Jette Frederiksen, Finn Sellebjerg

**Affiliations:** ^1^ Danish Multiple Sclerosis Center, Department of Neurology, Copenhagen University Hospital – Rigshospitalet, Glostrup, Denmark; ^2^ The Neurogenetics Clinic, Danish Dementia Research Center, Copenhagen University Hospital – Rigshospitalet, Copenhagen, Denmark; ^3^ Department of Clinical Medicine, University of Copenhagen, Copenhagen, Denmark

**Keywords:** early multiple sclerosis, CD20^+^ T cells, T cell-CNS cell interaction, blood-brain barrier, CNS immunosurveillance

## Abstract

Relapsing-remitting multiple sclerosis (RRMS) is a chronic immune-mediated disease of the central nervous system (CNS) characterized by episodic relapses of neurological symptoms. Early diagnosis and treatment of patients is key to prevent relapses and irreversible damage to the CNS. However, development of therapies targeting early MS is constrained by the lack of knowledge of early MS disease pathogenesis. In RRMS, nervous tissue damage is induced by CNS-infiltrating immune cells, including T cells. The aim of this study was therefore to investigate the early events of CNS infiltration by T cells in RRMS, including their interaction with astrocytes and brain endothelial cells. Using a human *in vitro* model system and tissue samples obtained from newly diagnosed patients and healthy individuals, we found that CD8^+^CD20^+^ T cells could cross the blood-brain-barrier (BBB) and blood-cerebrospinal fluid-barrier in the absence of inflammation. As CD8^+^CD20^+^ T cells are present in the CNS before inflammation and are highly enriched in MS lesions once the disease is initiated, they may be among the first effector cells in RRMS. Our study also showed that CD4^+^CD20^+^ T cells can stimulate brain endothelial cells to express adhesion molecules and chemokines needed for peripheral immune cells to adhere and migrate through the BBB, and stimulate astrocytes to produce chemokines directing transmigrating cells to the site of inflammation. This suggests that CD4^+^CD20^+^ T cells, once migrated to the CNS, strengthens the infiltration of immune cells to the CNS and hereby amplify CNS inflammation. Altogether, this indicates that CD20^+^ T cells play a role in the early phases of MS-inflammation and therefore are candidate targets for early treatment.

## Introduction

Multiple sclerosis (MS) is a chronic, incurable disease that affects the central nervous system (CNS) (1, 2). Most patients have a relapsing-remitting form of MS (RRMS) from onset, where T cells infiltrating the CNS are considered as major pathogenic effector cells (3–6). Patients benefit from early treatment to effectively reduce the number of relapses and to prevent irreversible damage to the CNS (7). However, MS often develops for years before symptoms reach a clinical threshold and the diagnosis is made (8, 9). During this prodromal or subclinical phase of MS, CNS inflammation and neuroaxonal damage is initiated, as demonstrated by elevated levels of the axonal injury biomarker neurofilament light chain (NFL) years before the clinical onset (10, 11). Additionally, our knowledge of early MS disease pathogenesis is limited, further restricting the opportunity for an optimal early treatment strategy.

A hallmark of RRMS is the formation of lesions within the CNS. Peripheral immune cells infiltrating the CNS through transmigration of the blood-brain-barrier (BBB) or the blood-cerebrospinal fluid-barriers (B-CSF-B) likely contribute to this lesion formation (3, 4, 12, 13). The BBB, also known as the neurovascular unit, consists of a tight network of cooperating cells, including endothelial cells, pericytes, and astrocytes (14). Brain endothelial cells line the cerebral microvessels and are the first cells to interact with peripheral immune cells intending to migrate into the CNS (15). Under steady state conditions, brain endothelial cells only express low levels of adhesion molecules important for immune cells to interact and move across the endothelial layer, including VCAM-1 and ICAM-1. However, these molecules are strongly upregulated following inflammation (15, 16). Besides adhesion molecules, peripheral leukocyte migration depends on chemokines produced by endothelial cells and activated astrocytes. Astrocytes, a primary neuron-supporting and BBB-controlling CNS cell, have a prominent role in resolution of active lesions in patients with MS once inflammation has subsided (17). Beyond this reparative role, astrocytes are now also recognized as important players in the very early phases of MS lesion formation. In support of this, animal studies have shown that astrocytes are activated prior to the CNS immune cell migration observed during relapses (18, 19). Activated astrocytes may contribute to inflammation in the early phases of MS by producing soluble factors that increase the permeability of the BBB and chemokines that attract peripheral immune cells (17–20). Interestingly, a recent study has suggested that CD4^+^ helper T cells of the Th1-phenotype and their effector cytokines can induce astrocyte production of chemokines, triggering infiltration of immune cells (20). We and others have previously shown that CD4^+^ T cells expressing CD20 are of a Th1-like phenotype and that CD20^+^ T cells are closely associated with the inflammation observed in patients with MS (21, 22). Therefore, it is possible that CD4^+^CD20^+^ T cells are implicated in the activation of astrocytes observed in patients with MS.

This study aimed to investigate the early events of immune activation seen in RRMS. This included migration of T cells across the BBB and the interplay between T cells and endothelial cells and astrocytes of the neurovascular unit.

## Materials and methods

### Study population, protocol approvals, and ethics

In this observational case-control study, four cohorts were included. The first cohort consisted of CSF cell samples from 12 healthy individuals, collected at the Neurogenetics Clinic, Danish Dementia Center at Rigshospitalet in Copenhagen. The second cohort consisted of CSF cell samples from 10 symptomatic control subjects and 16 patients with early RRMS; the third of blood samples from 29 healthy individuals and 41 patients with early RRMS; the fourth of cryopreserved peripheral blood mononuclear cell (PBMC) samples from 12 healthy individuals and 12 treatment naïve patients with RRMS, all collected at the Danish Multiple Sclerosis Center at Rigshospitalet. There was no significant difference in sex or age distribution between the groups in each cohort. Demographic characteristics of all cohorts are shown in **Supplementary Table 1**. Healthy individuals included had no neurological, autoimmune, or other chronic illness. Symptomatic control subjects were defined as patients with symptoms warranting a lumbar puncture but with no indication of inflammation or neurological disorder after diagnostic work-up (23). All MS patients were diagnosed with RRMS based on the 2017 McDonald criteria (24) and none had received methylprednisolone treatment within one month prior to sampling. Furthermore, patients with early RRMS were defined as treatment naïve patients with onset of disease within one year of sampling.

All participants gave informed, written consent to participation. The study was approved by the Ethics Committee of the Capital Region of Denmark (protocol number H2-2011-085, H-17005703, and H-16047666).

### Blood and CSF samples

Venous blood was collected, and PBMCs isolated by density gradient centrifugation using Lymphoprep (Axis-Shield, Oslo, Norway). The cells were washed twice in cold phosphate buffered saline (PBS)/2 mM ethylenediaminetetraacetic acid (EDTA) and either cryopreserved in fetal bovine serum (FBS; Thermo Fisher Scientific, MA, USA)/10% dimethyl sulfoxide (DMSO; Sigma-Aldrich, MO, US) at -150°C or directly applied to *ex vivo* flow cytometry or *in vitro* assays. CSF was collected in a 15 ml polypropylene tube on ice and immediately centrifuged for 10 min at 400 g at 4°C to separate cells from fluid, and the cells resuspended in PBS/2% FBS/0.02% sodium azide (NaN_3_; Sigma-Aldrich). CSF cells were applied to flow cytometry within one hour of sampling. CSF cell counts were measured during routine assessment with counts below the lower limit of quantification of 3 cells/µl truncated as 2 cells/µl.

### *Ex vivo* flow cytometric analysis

For *ex vivo* flow cytometry of freshly isolated PBMCs and CSF cells, cells were incubated in FcR-blocking reagent (Miltenyi Biotec, Bergisch Gladbach, Germany) to prevent nonspecific antibody binding, and thereafter stained in PBS/2% FBS/0.02% NaN_3_ with a combination of fluorochrome-conjugated antibodies for 25 min on ice. Cohort 1 cells were stained using Abs against (conjugate; clone): CD3 (APC-Cy7; HIT3a), CD4 (PerCP-Cy5.5; RPA-T4), CD20 (PE-Cy7; 2H7), CD183 (CXCR3, PE; G025H7), CD196 (CCR6, BV605; G034E3) all from BioLegend (CA, USA). Cohort 2: CD3 (BV421; OKT3), CD4 (PerCP; RPA-T4), CD8 (BV605; RPA-T8), CD20 (PE-Cy7; 2H7), all from BioLegend. Cohort 3: CD3 (BV421; OKT3), CD4 (PerCP; RPA-T4), CD8 (BV605; RPA-T8), CD20 (PE-Cy7; 2H7) from BioLegend, MT1-MMP (MMP14, APC; 128527) from R&D Systems (MN, USA), and MT5-MMP (MMP24, APC-Cy7; RM0289-7J71) from Novus Biologicals (CO, USA). Isotype-matched controls were used to correct for nonspecific antibody binding and spectral overlap where appropriate. Data were acquired on a FACSymphony or FACS Canto II flow cytometer (BD Biosciences) and data analysis performed using the software FlowJo (TreeStar, OR, USA).

### *In vitro* blood-brain-barrier model

The BBB model was assembled as previously described (25). In short, 3.0 µm pore polyester transwell inserts (CellQuart; SABEU, Northeim, Germany) were coated with 20 µg/ml human fibronectin on both sides (Sigma, Merck, MO, USA) at 37°C, 5% CO_2_ for 2 h. Hereafter, 95.000 human brain astrocytes/cm^2^ (Gibco, Waltham, MA, USA) in astrocyte medium (Gibco) were grown into a monolayer on the external side of the insert for 3 days and 95.000 human brain microvascular endothelial cells (HBMEC)/cm^2^ (PeloBiotech, Martinsried, Germany) in HBMEC-medium (PeloBiotech) on the internal side of the insert for an additional 2 days at 37°C, 5% CO_2._ The quality of the coculture was monitored by measuring the transendothelial electrical resistance (TEER-value). Transmigration experiments were performed when stable resistance measurements indicated a cell layer forming a barrier, i.e., a TEER-value of 40 Ω×cm2 when background was subtracted. 700.000 purified human primary T cells in RPMI (Gibco) from 12 healthy individuals and 12 newly diagnosed patients with RRMS were transferred to the upper compartment and left to migrate for 5 h at 37°C, 5% CO_2_. For this, cryopreserved PBMCs were used, and T cells purified with a human T cell isolation kit from Stem Cell Technologies (Vancouver, Canada). To the lower compartments RPMI supplemented with B-27 (Gibco) was added with or without 1000 ng/ml CXCL10 (R&D systems) as a chemoattractant. To induce inflammation of the BBB, 100 U/ml TNF-α and IFN-γ (R&D Systems, MN, USA) was added to the upper compartment 24 h prior to performing the migration assay. After 5 h of migration, a standardized amount of flow count fluorospheres (Beckman Coulter, CA, USA) was added to the lower compartment of each well. Migrated cells plus flow count-fluorospheres were harvested, stained for flow cytometry as described above using fluorochrome-conjugated antibodies against CD3 (AF488; UCHT1), CD4 (PerCP; SK3), CD8 (BV421; RPA-T8) and CD20 (PE/Cy7; 2H7) all from BioLegend, and the number and percent of migrated cells calculated as:


$$Number\;of\;migrated\;cells=\;\frac{detected\;number\;of\;cells\;\times \;total\;number\;of\;count-fluorospheres\;added}{detected\;number\;of\;count-fluorospheres}$$

$$Percent\;migrated\;cells=\frac{number\;of\;migrated\;cells}{number\;of\;cells\;in\;the\;control\;well}\times 100$$

### CD4^+^ T cell conditioned medium

To generate CD4^+^ T cell conditioned medium PBMCs were purified from 80 ml blood, as described above. T cells were hereafter negatively isolated using a human T cell isolation kit from Stem Cell Technologies, stained with fluorochrome-conjugated antibodies against αβTCR (APC; IP26), CD4 (PerCP; SK3), and CD20 (PE-Cy7; 2H7) all from BioLegend and subjected to fluorescence-activated cell sorting on a BD Melody FACS (BD Biosciences). Sorted CD4^+^CD20^+^ and CD4^+^CD20^-^ T cells were then stimulated for 20 h in flat-bottom plates with 4 µg/ml anti-CD3 (OKT3; BioLegend) and 1 µg/ml anti-CD28 (CD28.2; BioLegend) antibodies bound to the plate. The supernatants, i.e., CD4^+^ T cell-conditioned medium, were frozen at -80°C until use.

### RNA-array measurement of stimulated HBMECs

90.000 HBMECs/cm^2^ in HBMEC-medium were plated in 24 well-plates coated with 20 µg/ml human fibronectin for 48 h. Hereafter, the medium was changed to HBMEC medium containing 12% RPMI, 12% CD4^+^CD20^+^ or CD4^+^CD20^-^ T cell-conditioned medium and incubated for 24 h. Then, HBMECs were lyzed in Qiazol lysis buffer (QIAGEN, Hilden, Germany), chloroform added, and samples separated into an aqueous and an organic phase by centrifugation. RNA was precipitated from the aqueous phase using an RNeasy kit from Qiagen and used for Affymetrix analysis. Affymetrix analysis was performed at the Core facility for Genomic Medicine, the Kennedy Center, Rigshospitalet, Denmark, according to the manufacturer. The array used was the Human Gene 2.0 ST Array.

### Upregulation of adhesion molecules on HBMECs

90.000 HBMECs/cm^2^ in HBMEC-medium were plated in 48 well-plates coated with 20 µg/ml human fibronectin for 48 h, and medium changed to HBMEC-medium containing 12% RPMI, 12% CD4^+^CD20^+^ or CD4^+^CD20^-^ T cell conditioned medium. After 24 h at 37°C, 5% CO_2_, medium was removed, cells washed in PBS and fixated with 4% paraformaldehyde (PFA)/PBS (BioLegend) at RT for 20 min. The cells were then washed twice in PBS, blocked in 5% FBS/PBS for 30 min at RT, and incubated for 3 h at RT in 10 µg/ml mouse anti-CD106 (VCAM-1; STA, BioLegend) or sheep anti-CD54 (ICAM-1; R&D Systems) antibodies in 5% FBS/PBS. The cells were washed twice in PBS, incubated for 1 h at RT with 10 µg/ml polyclonal donkey anti-mouse IgG-AF594 or donkey anti-sheep IgG-AF594 (Invitrogen, MA, USA), washed again, and anti-fade/DAPI-solution (Invitrogen) added. Images were acquired using an EVOS microscope (Invitrogen).

90.000 HBMECs/cm^2^ in HBMEC-medium were further plated in 48 well plates and stimulated with various concentrations of RPMI, CD4^+^CD20^+^ and CD4^+^CD20^-^ T cell-conditioned medium. After 24 h, HBMECs were detached from the plates using Accutase (Sigma-Aldrich) and cells stained with fluorescence-conjugated antibodies against VCAM-1 (PE; STA), ICAM-1 (AF647; HA58), HLA-DR (BV421; L243), HLA-A,B,C (APC-Cy7; W6/32) and the endothelial cell marker CD31(BV605; WM59), all from BioLegend. Propidium iodide was included as a cell death marker, and cell surface levels of target molecules analyzed by flow cytometry.

### Astrocyte production of chemokines

90.000 astrocytes/cm^2^ in astrocyte-medium were plated in 96 well-plates coated with 20 µg/ml human fibronectin for 48 h. The medium was then changed to astrocyte medium containing various concentrations of RPMI, CD4^+^CD20^+^, or CD4^+^CD20^-^ T cell conditioned medium. After 24 h incubation at 37°C, 5% CO_2_, the medium was removed, astrocytes washed, and fresh astrocyte medium applied. After an additional 24 h incubation, the supernatant was applied to ELISA. ELISA measurements included CCL5, CXCL9, CXCL10, and CXCL11, all from R&D Systems.

### Statistical analysis

For analysis of sex differences between groups a Chi-square test was performed, and for analysis of age differences between groups a Mann-Whitney U test was applied. To compare cell frequencies or numbers between two groups a Mann-Whitney U test was used, and to compare cell frequencies or numbers within groups, a Wilcoxon matched-pairs rank test was used. P < 0.05 was considered statistically significant.

### Data availability

Data are available in anonymized form and can be shared by request from any qualified investigator. Sharing requires approval of a data transfer agreement in accordance with GDPR and Danish data protection regulation.

## Results

### CD8^+^CD20^+^ T cells can cross the non-inflamed blood-brain barrier

**Figure 1:**
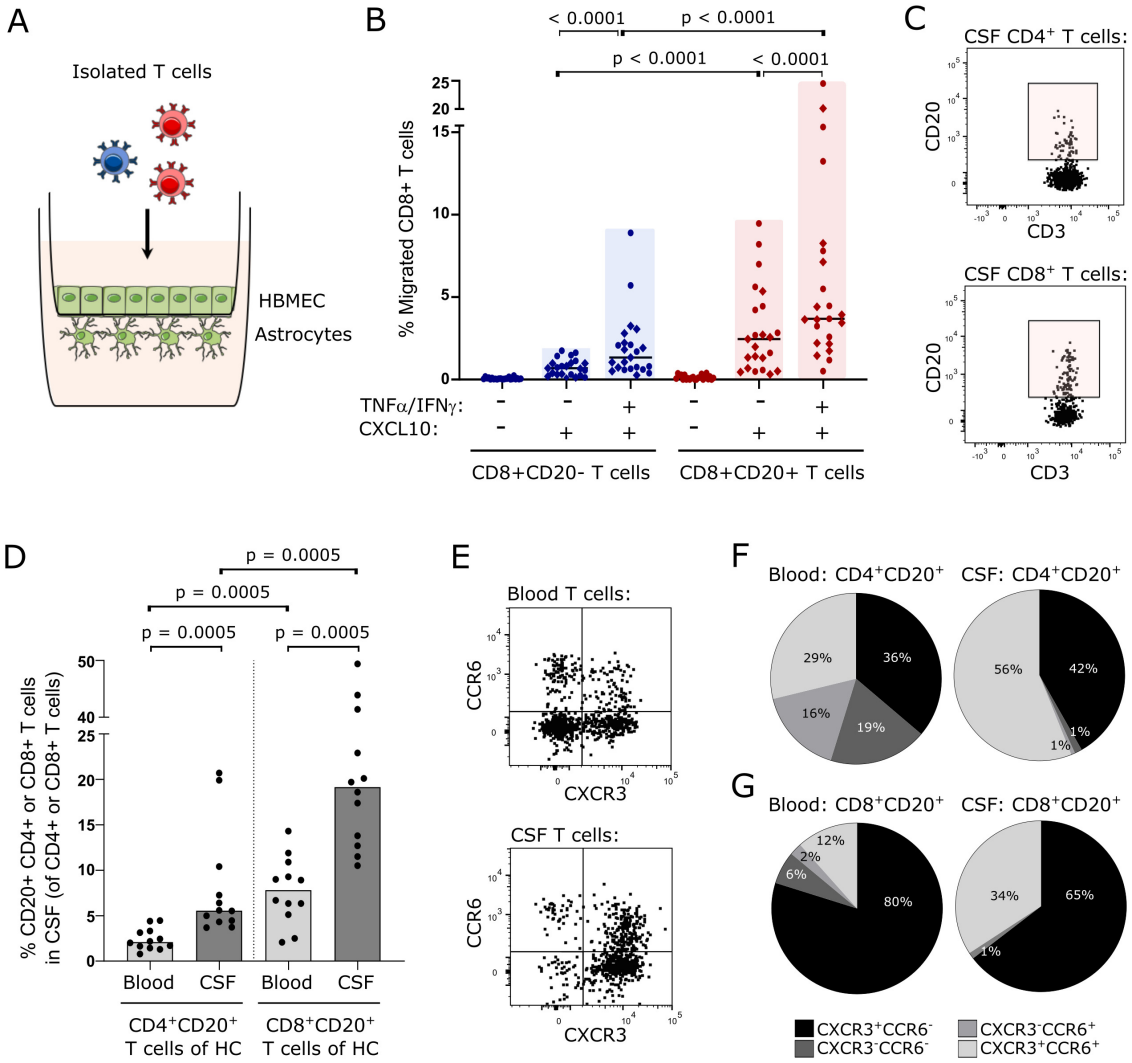
CD8^+^CD20^+^ T cells can cross the uninflamed blood-brain-barrier. **(A)** Schematic illustration of the *in vitro* human BBB-model used. The model is based on a Boyden-chamber assay with human primary astrocytes grown on the abluminal side and human brain microvascular endothelial cells (HBMEC) on the luminal side of the membrane. **(B)** Percent migrated CD8^+^CD20^-^ and CD8^+^CD20^+^ T cells. Healthy individuals are indicated with circles and untreated patients with RRMS with diamonds. Median values are shown for all groups. Significant p-values are shown for all comparisons except for the two control groups (-TNFα/IFNγ -CXCL10) where no migration is observed. **(C)** Flow cytometry dot plot examples showing CD4^+^CD20^+^ T cells (upper panel) and CD8^+^CD20^+^ T cells (lower panel) in CSF of healthy individuals. **(D)** Frequency of CD4^+^CD20^+^ and CD8^+^CD20^+^ T cells in the blood and CSF of 12 healthy individuals. Median values are shown. **(E)** Flow cytometry dot plot examples showing CXCR3 and CCR6 staining of T cells in the blood (upper panel) and CSF (lower panel). **(F, G)**. Distribution of CXCR3^+^CCR6^-^, CXCR3^-^CCR6^+^, CXCR3^+^CCR6^+^, and CXCR3^-^CCR6^-^ T cells in the blood and CSF of the CD4^+^ **(F)** and CD8^+^ **(G)** T cells from healthy individuals. Mean values are shown.

Using an *in vitro* human BBB model, we analyzed trans-migration of human primarily CD20^-^ and CD20^+^ T cells under inflamed and non-inflamed conditions (**Figure 1A**). An inflamed BBB was achieved by a 24 h pre-incubation with IFNγ plus TNFα prior to migration. The chemoattractant CXCL10 was used to induce migration of T cells across the barrier. When purified T cells were left to cross the BBB, we found that T cells from healthy individuals (n=12) and untreated patients with RRMS (n=12) had an equal migration potential (data not shown). To increase power, data from healthy individuals and untreated patients with RRMS was pooled for further analysis. This showed that CD8^+^CD20^+^ T cells had a higher migration potential than the corresponding CD8^+^CD20^-^ T cells (**Figure 1B**; p<0.0001). Interestingly, we also observed that CD8^+^CD20^+^ T cells, in contrast to CD8^+^CD20^-^ T cells, had a high capacity to cross a non-inflamed BBB (**Figure 1B**; p<0.0001). Furthermore, we found a considerably higher frequency of CD8^+^CD20^+^ T cells crossing the non-inflamed BBB than CD4^+^CD20^+^ T cells (p = 0.0075; data not shown). The level of migration across the non-inflamed BBB barrier of CD4^+^CD20^+^ and CD4^+^CD20^-^ T cells was equal (p=0.15; data not shown). Also, migration across the inflamed BBB was higher in the CD8^+^CD20^+^ T cell population compared to CD4^+^CD20^+^ T cells (p = 0.023; data not shown).

### Intrathecal CD20^+^ T cells in CSF of healthy individuals

Considering the equal migration capacity of CD20^+^ T cells of healthy individuals and untreated patients with RRMS in our *in vitro* model, we next investigated if CD20^+^ T cells were present in the CSF of healthy individuals (n=12). Here, we found that 5.6% (4.4, 9.6) of CD4^+^ T cells in the CSF were CD20^+^ and 19.5% (13, 37) of CD8^+^ T cells were CD20^+^ (**Figures 1C, D**); median value (Q1, Q3). This observation shows that CD20^+^ T cells from healthy individuals can cross the B-CSF-B and further shows a clear predominance of CD8^+^CD20^+^ T cells compared to CD4^+^CD20^+^ T cells in the CSF (**Figures 1C, D**; p = 0.0005). We also observed that CD4^+^CD20^+^ and CD8^+^CD20^+^ T cells were enriched in the CSF compared to the blood (**Figure 1D**; p = 0.0005).

To analyze if CSF CD20^+^ T cells from healthy individuals had a proinflammatory phenotype, as previously described for CD20^+^ T cells from patients with RRMS (21), we measured the surface expression of CXCR3 and CCR6 (**Figure 1E**). This showed that 42% of CSF CD4^+^CD20^+^ T cells were Th1-like cells (CXCR3^+^CCR6^-^) and 56% Th17.1-like cells (CXCR3^+^CCR6^+^) (**Figure 1F**). Likewise, we found a distribution of CSF CD8^+^CD20^+^ T cells of 65% Tc1-like cells (CXCR3^+^CCR6^-^) and 34% Tc17.1-like cells (CXCR3^+^CCR6^+^) (**Figure 1G**). For comparison, the distribution of CXCR3/CCR6-expressing CD20^-^ T cells are shown in **Supplementary Figure 1**.

### Prevalence of intrathecal CD20^+^ T cells in the early disease course of patients with RRMS

**Figure 2:**
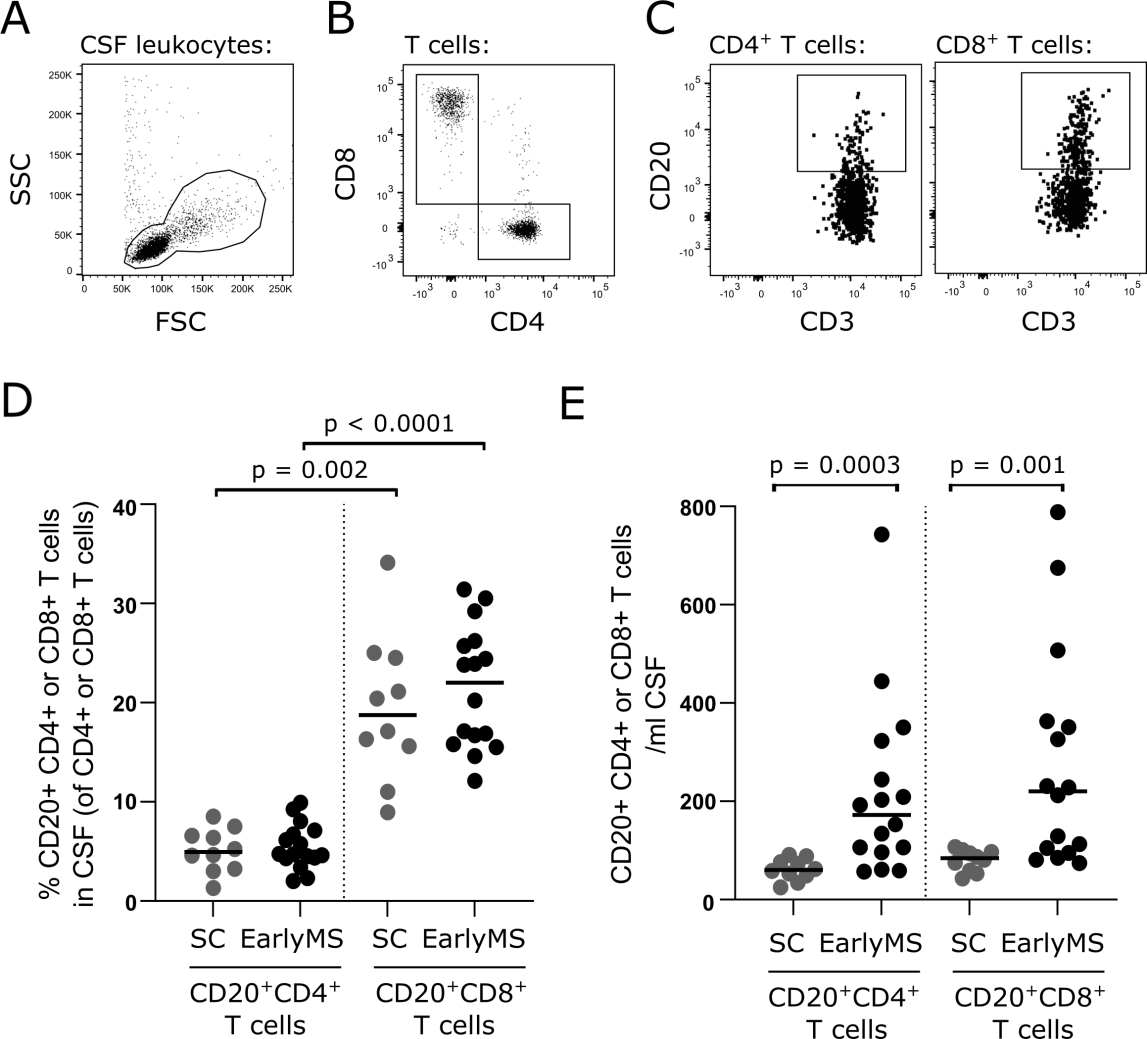
Intrathecal CD20^+^ T cells in patients with early MS. **(A–C)** Gating strategy of CD20^+^ T cells in CSF of 10 symptomatic controls (SC) and 16 patients with early RRMS (EarlyMS): leukocytes were defined in an FSC/SSC dot plot **(A)**, T cells gated according to CD3 expression, T cells subdivided into CD4^+^ and CD8^+^ T cells **(B)**, and CD20^+^ gate applied **(C)**. **(D, E)** Frequency and absolute number of CD4^+^CD20^+^ and CD8^+^CD20^+^ T cells in CSF. Median value is shown for all groups analyzed.

To validate that CD20^+^ T cells can be recruited to the CSF without CNS inflammation, we included a second cohort of 10 symptomatic controls with no inflammatory or neurological disease. Also, we included 16 patients with early MS (i.e., treatment-naïve and clinical disease onset within one year prior to sampling) to examine whether the prevalence of CSF CD20^+^ T cells in the early disease course of RRMS was increased compared to a non-inflammatory control environment. An example of CD20^+^ T cell identification is shown in **Figures 2A–C**. This analysis showed equal frequencies of CSF CD4^+^CD20^+^ T cells in symptomatic controls and patients with early MS, and likewise of CSF CD8^+^CD20^+^ T cells in the two groups (**Figure 2D**). However, the frequency of CSF CD8^+^CD20^+^ T cells was much higher than CSF CD4^+^ CD20^+^ T cells both in symptomatic controls (19% vs. 4.9%; p=0.002) and patients with early MS (22% vs. 4.7%; p<0.0001), **Figure 2D**. Due to pleocytosis in patients with early MS the absolute numbers of CD4^+^CD20^+^ T cells and CD8^+^CD20^+^ T cells were significantly higher in patients with early MS compared to symptomatic controls (p=0.0003 and p=0.001, respectively), **Figure 2E**.

### Increased expression of membrane-anchored metalloproteinases on peripheral CD20^+^ T cells

**Figure 3:**
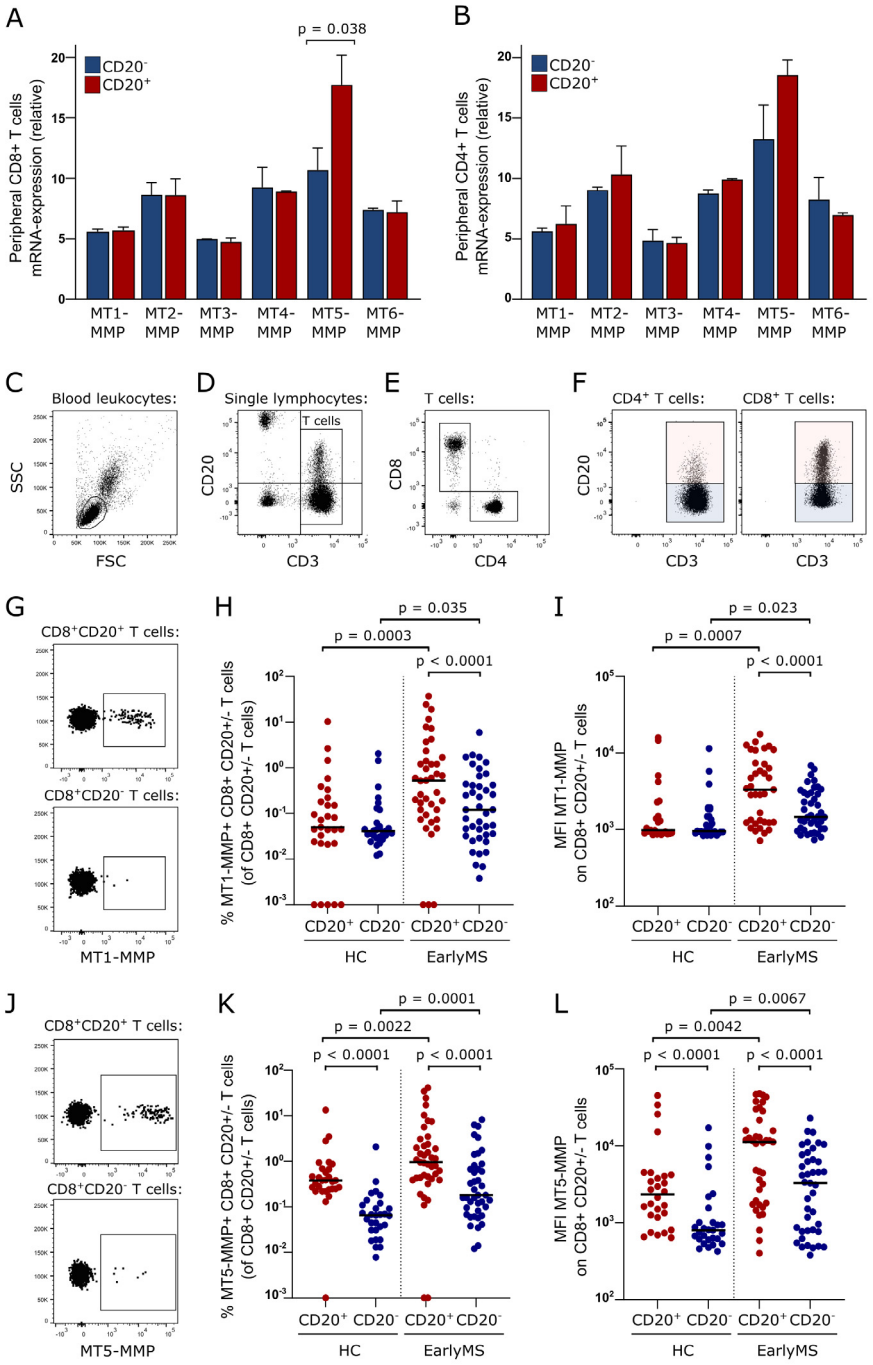
MT-MMP expression on CD8^+^CD20^+^ T cells. **(A, B)** Affymetrix analysis of MT-MMP expression in CD8^+^ T cells **(A)** and CD4^+^ T cells **(B)**. **(C–G)** Gating strategy of MT1-MMP^+^ and MT5-MMP^+^ CD20^+^ T cells in the blood of 29 healthy individuals (HC) and 41 patients with early RRMS (EarlyMS): lymphocytes were defined in a FSC/SSC dot plot **(C)**, T cells gated according to CD3 expression and CD20^+^ T cells defined **(D)**, T cells subdivided into CD4^+^ and CD8^+^ T cells **(E)**, a CD20^+^ and a CD20^-^ gate applied to each subdivision **(F)**, and MT1-MMP^+^ **(G)** or MT5-MMP^+^ **(J)** cells identified. **(H)** Frequency of MT1-MMP^+^ CD20^+^ and CD20^-^ CD8^+^ T cells. **(I)** Mean fluorescence intensity (MFI) of MT1-MMP expression on CD20^+^ and CD20^-^ CD8^+^ T cells. **(K)** Frequency of MT5-MMP^+^ CD20^+^ and CD20^-^ CD8^+^ T cells. **(L)**. MFI of MT5-MMP expression on CD20^+^ and CD20^-^ CD8^+^ T cells. Median value is shown for all groups analyzed.

To find a possible cause for the increased ability of blood CD20^+^ T cells, particularly CD8^+^CD20^+^ T cells, to cross the BBB, we explored a previously performed RNA microarray of peripheral CD20^+^ and CD20^-^ T cells (26) for the expression of membrane-anchored matrix metalloproteinases (MT-MMP). There are six known MT-MMPs in humans that potentially function to aid leukocytes in crossing endothelial cell layers (27). Analyzing the expression pattern of all six showed an increase in MT5-MMP in CD8^+^CD20^+^ T cells compared to CD8^+^CD20^-^ T cells of healthy individuals (**Figure 3A**). Although a similar tendency was observed in CD4^+^ T cells, this was not statistically significant (**Figure 3B**).

Cell surface expression of MT5-MMP together with MT1-MMP, which is a key regulator of cell migration (28), was subsequently measured on freshly isolated peripheral T cells from 29 healthy individuals and 41 patients with early MS. An example of CD20^+^ T cell identification is shown in **Figures 3C–F**. This showed an increased frequency and surface level of expression (MFI) of both MT1-MMP and MT5-MMP on CD8^+^CD20^+^ T cells compared to CD8^+^CD20^-^ T cells in patients with early MS. Also, both frequency and MFI of MT1-MMP and MT5-MMP was higher on CD8^+^CD20^+^ T cells from patients with early MS compared to healthy individuals (**Figures 3G–L**). The same was found when analyzing CD4^+^ T cells (**Supplementary Figure 2**). Interestingly, when comparing CD4^+^CD20^+^ and CD8^+^CD20^+^ T cells in patients with early MS, MT1-MMP and MT5-MMP expression and MFI were significantly higher on CD4^+^CD20^+^ T cells (all p<0.0001). Of note, more T cells expressed MT5-MMP than MT1-MMP, and all MT1-MMP^+^ T cells co-expressed MT5-MMP at a high surface level.

### CD4^+^CD20^+^ T cells upregulate VCAM-1 and ICAM-1 on human brain endothelial cells

**Figure 4:**
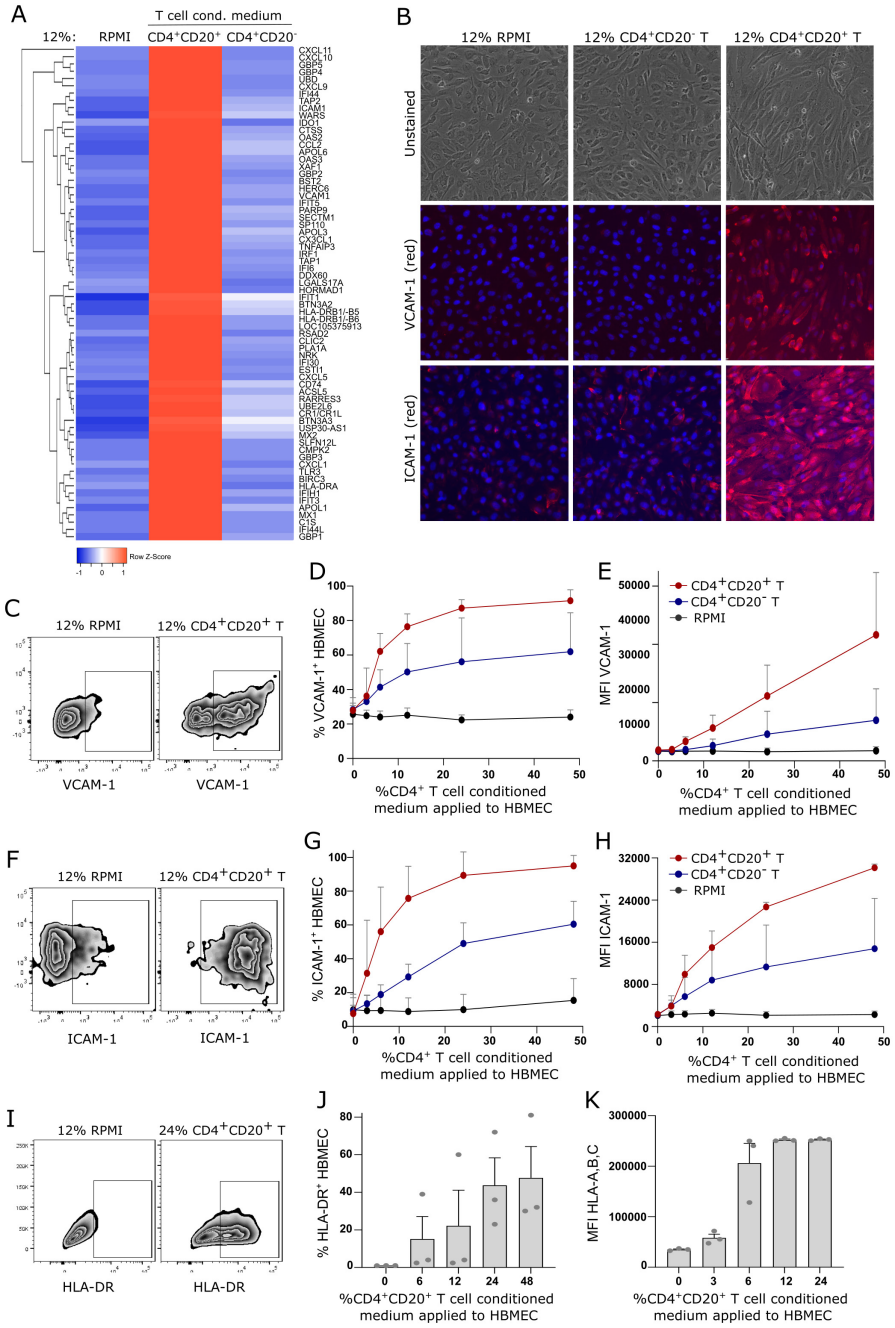
CD4^+^CD20^+^ T cells induce upregulation of VCAM-1, ICAM-1, and MHC on HBMECs. **(A)** Heat map of affymetrix microarray data of human brain microvascular endothelial cells (HBMECs) stimulated for 24 h in 12% RPMI, CD4^+^CD20^+^ or CD4^+^CD20^-^ T cell conditioned medium. **(B)** Fluorescence microscopy of HBMECs stimulated for 24 h in 12% RPMI, CD4^+^CD20^+^ or CD4^+^CD20^-^ T cell conditioned medium. DAPI stain (blue): nucleus; red stain (middle row): VCAM-1; red stain (lower row): ICAM-1. **(C–E)** Flow cytometry dot plot example of VCAM-1 expression on HBMECs **(C)**, frequency of VCAM-1^+^ HBMECs **(D)**, and VCAM-1 mean fluorescence intensity (MFI) **(E)**. **(F–H)**. Flow cytometry dot plot example of ICAM-1 expression on HBMECs **(F)**, frequency of ICAM-1^+^ HBMECs **(G)**, and ICAM-1 MFI **(H)**. **(I, J)** Flow cytometry dot plot example of HLA-DR expression on HBMECs **(I)**, and frequency of HLA-DR^+^ HBMECs **(J)**. **(K)** MFI of HLA-A,B,C expression on HBMECs. The plots shown are mean-values of three independent experiments using conditioned medium from three different individuals.

Peripheral CD8^+^CD20^+^ T cells that cross the BBB upon inflammation are expected to participate in cytotoxic events, whereas CD4^+^CD20^+^ T cells likely perform supportive functions of other cells in the CNS. To investigate what supportive functions CD4^+^CD20^+^ T cells may perform during inflammation, CD4^+^CD20^+^ and CD4^+^CD20^-^ T cells were stimulated for 24 h with a polyclonal stimulus in RPMI to generate T cell-conditioned medium of primed CD4^+^CD20^+^ and CD4^+^CD20^-^ T cells. Hereafter, HBMECs were cultured for 24 h in HBMEC medium containing 12% RPMI, 12% CD4^+^CD20^+^ or CD4^+^CD20^-^ T cell conditioned medium and the HBMECs subjected to a DNA-array analysis. This analysis showed a pronounced increase in mRNA expression of various genes when HBMECs were cultured in CD4^+^CD20^+^ T cell conditioned medium compared to both RPMI and CD4^+^CD20^-^ T cell conditioned medium. Of the 68 top hits shown in **Figure 4A**, we found upregulation of a range of mRNA encoded by genes involved in anti-viral defense, including interferon-inducible genes (IFIs), myxovirus resistance genes (MX1-2), and genes encoding guanylate binding proteins (GBP1-5). In addition, we observed upregulation of C-X-C motif chemokines known to attract peripheral immune cells, including CXCL1, CXCL5, CXCL9, CXCL10, and CXCL11. Interestingly, we also found increased transcription of genes encoding VCAM-1 (vascular cell adhesion molecule 1) and ICAM-1 (intercellular adhesion molecule 1), which are both essential adhesion molecules for the migration of peripheral immune cells to the CNS.

To support the observation of increased VCAM-1 and ICAM-1 mRNA, we used fluorescence microscopy to evaluate protein expression of both adhesion molecules on HBMECs in response to CD4^+^CD20^+^ T cell conditioned medium. This showed a clear upregulation of both VCAM-1 and ICAM-1 (**Figure 4B**). We next validated this finding by flow cytometry of HBMECs cultured in increasing dosages of RPMI and CD4^+^ T cell conditioned medium (**Figures 4C–H**). This showed increased frequencies and expression level of VCAM-1^+^ and ICAM-1^+^ HBMECs with increasing concentrations of CD4^+^CD20^+^ T cell conditioned medium. The plots shown are mean values of three independent experiments using conditioned medium from three individuals.

### CD4^+^CD20^+^ T cells induce expression of MHC on human brain endothelial cells

Presentation of myelin antigens, derived from internalized and processed myelin debris, on MHC class II on brain endothelial cells during inflammation has been shown to promote recruitment of antigen-specific T cells to the CNS (29). Culturing HBMECs in CD4^+^CD20^+^ T cell conditioned medium for 24 h led to an increase in HLA-DR mRNA (RNA-array, **Figure 4A**) and an induction of HLA-DR protein (MHC class II) on HBMECs (**Figures 4I, J**). This suggests a role for CD4^+^CD20^+^ T cells in the recruitment of antigen-specific T cells to the CNS during inflammation. While all HBMECs expressed MHC class I, exposure to CD4^+^CD20^+^ T cell conditioned medium for 24 h increased the surface expression level of HLA-A, B and C (MHC class I), **Figure 4K**.

### CD4^+^CD20^+^ T cells induce astrocyte production of CCL5, CXCL9, CXCL10 and CXCL11

**Figure 5:**
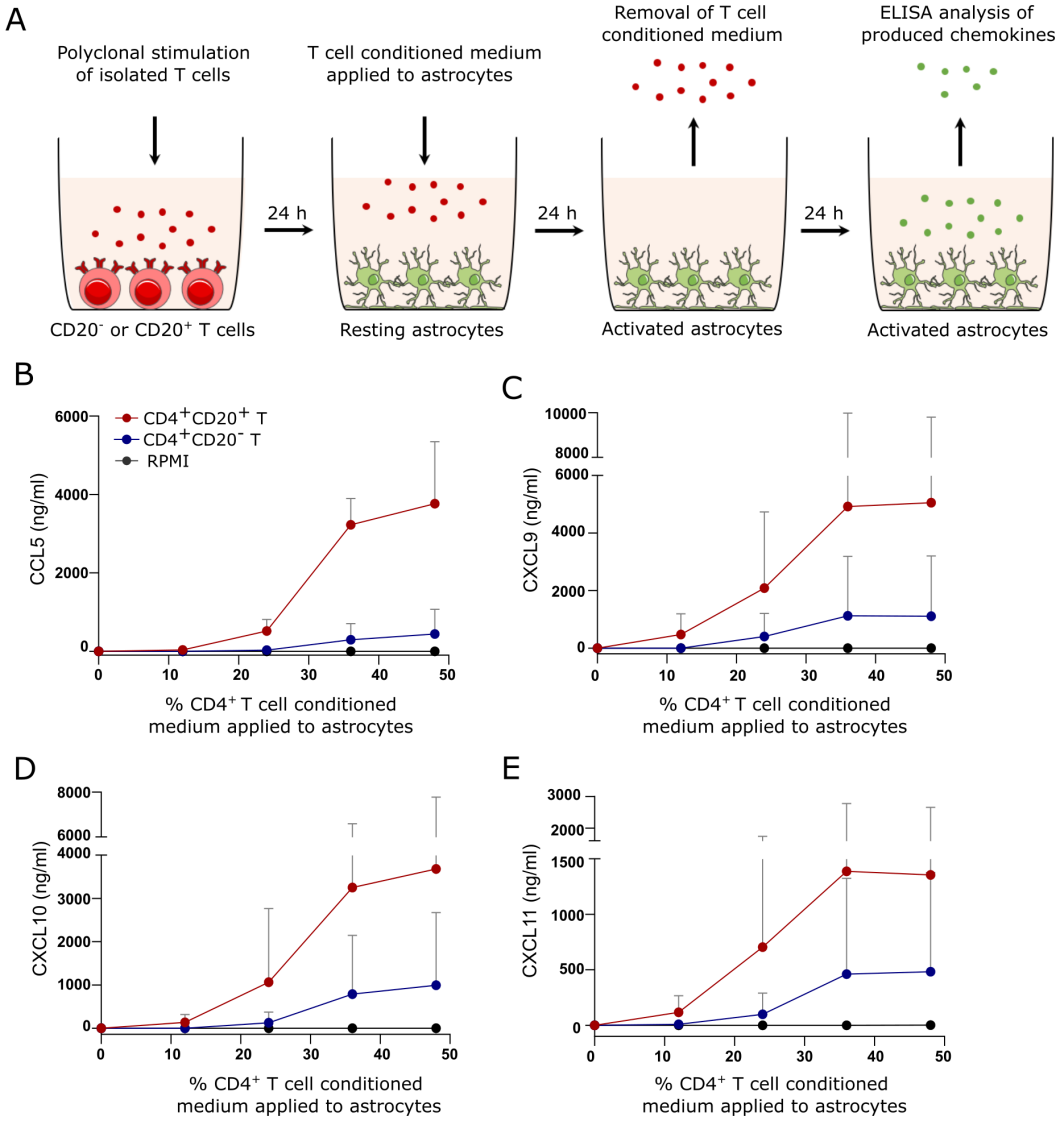
CD4^+^CD20^+^ T cells induce production of CCL5, CXCL9, CXCL10, CXCL11 in human astrocytes. **(A)** Schematic illustration of the astrocyte culture setup. **(B–E)**. Concentration of CCL5 **(B)**, CXCL9 **(C)**, CXCL10 **(D)**, and CXCL11 **(E)** produced by astrocytes cultured in RPMI, CD4^+^CD20^+^ or CD4^+^CD20^-^ T cell conditioned medium. The plots shown are mean-values of three independent experiments using conditioned medium from a total of four different individuals.

In the early stages of MS, astrocytes are present at the lesion areas, producing chemokines to chemoattract immune cells (18–20). To further investigate a possible role of helper CD4^+^CD20^+^ T cells in CNS during inflammation, we investigated whether activated CD4^+^CD20^+^ T cells could contribute to astrocyte chemokine production. For this, resting astrocytes were cultured for 24 h in various concentrations of CD4^+^CD20^+^ and CD4^+^CD20^-^ T cell-conditioned medium. The medium was hereafter removed, the cells were washed and subsequently incubated for an additional 24 h. Chemokine production by the astrocytes was then analyzed by ELISA. A schematic illustration is shown in **Figure 5A**. CXCL9, CXCL10, and CXCL11 were chosen as targets as they represent chemoattractants for cells expressing CXCR3 which includes the majority of T cells found intrathecally in humans, and as CXCL10 is increased in the CSF of patients with MS (30–32). CCL5 was included in the analysis as a previous study of human primary astrocytes pinpointed this chemokine as the most prominently expressed upon astrocyte activation (33) and CCL5 is elevated in the CSF of patients with MS (31, 34). Data from the ELISA experiments showed a substantial increase in CCL5, CXCL9, CXCL10, and CXCL11 produced by astrocytes in response to CD4^+^CD20^+^ T cell conditioned medium (**Figures 5B–E**). CCL5, CXCL9, CXCL10, and CXCL11 production was also induced by astrocytes cultured in CD4^+^CD20^-^ T cell conditioned medium, however, at a much lower concentration (**Figures 5B–E**). Due to the enhanced ability of CD8^+^CD20^+^ T cells to cross the BBB, we also analyzed if CD20^+^CD8^+^ T cells could induce cytokine production in astrocytes as observed with CD4^+^CD20^+^ T cells. This showed that astrocytes produced both CXCL10 and CXCL11 in response to CD8^+^CD20^+^ T cell-conditioned medium, and that they did not induce CCL5 production (**Supplementary Figure 3**). The plots shown are mean values of three independent experiments using a conditioned medium from a total of four individuals.

## Discussion

Understanding how immune cells contribute to the development of MS is far from complete. An inside-out model suggests that damage to oligodendrocytes and myelin is an initial event, potentially occurring years before onset of symptoms (35). This damage may be caused by an infection or trauma, facilitating microglia activation and the release of myelin antigens to the periphery followed by activation and recruitment of myelin-specific autoreactive T cells from the blood (4, 35). Previously, we have shown that CD20^+^ T cells are highly reactive to myelin antigens and have an increased migration potential compared to CD20^-^ T cells (21). In the present study, we used an *in vitro* human BBB-model based on a coculture of human brain endothelial cells and astrocytes and found that CD8^+^CD20^+^ T cells had an increased ability to migrate across the BBB during inflammation compared to CD8^+^CD20^-^ T cells and CD4^+^ T cells. This is in line with a study showing an enrichment of CD8^+^CD20^+^ T cells in white matter lesions of patients with MS (36). Additionally, we observed that CD8^+^CD20^+^ T cells had the ability to cross the BBB in the absence of inflammation, regardless of whether they originated from healthy individuals or patients with MS. This observation is in consistency with mouse studies showing that certain T cells can cross the brain barriers in the absence of neuroinflammation as part of CNS immunosurveillance (15). To strengthen the hypothesis that CD8^+^CD20^+^ T cells are involved in CNS immunosurveillance, we analyzed CSF T cells *ex vivo* from healthy individuals. Consistent with our observation in the BBB model, this also showed an enrichment of CSF CD8^+^CD20^+^ T cells. T cells can enter the CNS either by crossing the BBB through the perivascular space or the B-CSF-B through the subarachnoid or ventricular spaces. These regions are border compartments, allowing further migration of T cells into the brain parenchyma only in response to a threat, such as infectious agents (37). Our observations from the BBB model and the CSF of healthy individuals therefore represents migration of T cells to two different CNS compartments, but altogether suggests that human CD8^+^CD20^+^ T cells may be involved in immunosurveillance of the CNS. The immunological profile of CD8^+^CD20^+^ T cells as protectors against viral infections (38, 39) and tumor cells (40, 41) is in coherence with a possible role of these cells in CNS immunosurveillance. One could speculate that activation and recruitment of CNS border-patrolling CD8^+^CD20^+^ T cells into the brain parenchyma to fight a pathogen could lead to the release of myelin antigens to the periphery, inducing a domino effect leading to neuroinflammation and injury, as proposed by the inside-out model of MS.

In search of cell characteristics that could account for the superior ability of CD8^+^CD20^+^ T cells to cross the BBB, we measured the expression of the membrane-anchored matrix metalloproteinases MT1-MMP (MMP14) and MT5-MMP (MMP24) on peripheral T cells. MT-MMPs degrade various components of extracellular matrix, including collagen, laminin, fibronectin, and fibrinogen, thereby potentially facilitating cell migration (27, 28). Our analysis showed that MT1-MMP and MT5-MMP expression and MFI were higher on CD8^+^CD20^+^ T cells than CD8^+^CD20^-^ T cells and more pronounced in patients with MS than controls. However, only a minor population of blood CD8^+^CD20^+^ T cells expressed MT-MMPs, and expression of both MT1-MMP and MT5-MMP was significantly higher on CD4^+^CD20^+^ T cells compared to CD8^+^CD20^+^ T cells. Therefore, although MT-MMPs may participate in BBB-migration of CD8^+^CD20^+^ T cells, they are likely not the primary drivers of this process.

To investigate if inflammation in the early stages of MS increased the recruitment of CD20^+^ T cells to the CSF following clinical onset of disease, we analyzed CSF cells from a cohort of treatment-naïve patients sampled within one year of clinical onset of disease. Individuals without intrathecal inflammation or neurological disease were included as controls. This analysis showed a strong increase in the absolute numbers of CSF CD4^+^CD20^+^ and CD8^+^CD20^+^ T cells in early MS compared with controls.

Despite the many fold higher number of CD20^+^ T cells in CSF of patients with MS, the frequency of T cells expressing CD20 was similar to controls. This may be surprising as the frequency of CD20^+^ T cells in blood is higher in patients with RRMS (21) and since these patients concomitantly have an increased CSF level of CXCL10 (31, 32), which has the potential to attract CXCR3-expressing cells. CD20^+^ T cells have a higher surface expression of CXCR3 than CD20^-^ T cells (21), and given the higher level of both CD20^+^ T cells to be attracted and chemoattractant in patients with MS, a higher frequency in the CSF would be expected. Although not statistically significant, there was a numerically higher frequency of CSF CD8^+^CD20^+^ T cells in patients with early MS compared to controls. Additionally, our observation likely reflects the pool of CD20^+^ T cells residing at the CNS borders and not the CD20^+^ T cells further recruited to the brain parenchyma. A previous study described an enrichment of CD8^+^CD20^+^ T cells in white matter lesions of patients with MS (36), emphasizing the idea that myelin-specific CD8^+^CD20^+^ T cells may largely be recruited to the brain parenchyma during inflammation in patients with MS and not reside at the CNS borders. In a recent study, we showed that the prevalence of CD8^+^CD20^+^ T cells in CSF of patients with primary progressive MS was associated with the concentration of myelin-debris in the form of myelin basic protein (MBP) and development of new T2-weighted lesions (42). Likewise, we have shown that the prevalence of CD20^+^ T cells in the CSF of patients with RRMS correlated with MBP concentrations and disease severity (21). Therefore, our observation of an increased number of recruited CD20^+^ T cells in patients with early MS, together with findings in the literature suggesting a clinical impact of this recruitment, implies that CD20^+^ T cells likely participate in the maintenance of CNS inflammation.

Whereas reactivated CD8^+^CD20^+^ T cells at the CNS borders are expected to enter the CNS parenchyma and participate in nervous tissue damage in patients with MS, we questioned what function border-patrolling CD4^+^CD20^+^ T cells were committed to upon activation. CD4^+^ T cells are helper T cells generally involved in supporting the function of adjacent cells. Therefore, we generated CD4^+^CD20^+^ T cell conditioned medium and applied it to cultures of brain endothelial cells and astrocytes as these are cells of the neurovascular unit likely influenced by activated CD4^+^CD20^+^ T cells. An RNA-array of brain endothelial cells revealed that CD4^+^CD20^+^ T cell-conditioned medium led to a substantial change in many pathways. This included promotion of a strong anti-viral state through the expression of genes that participate in the cellular antiviral response, increased transcription of genes encoding a long list of chemokines known to attract peripheral immune cells including CXCL9, CXCL10 and CXCL11, and genes encoding the adhesion molecules VCAM-1 and ICAM-1 which are essential for immune cell interaction with the endothelial cells of the BBB (15). Due to the importance of endothelial upregulation of VCAM-1 and ICAM-1 for the CNS-recruitment of immune cells during inflammation, we validated this finding by fluorescence microscopy and flow cytometry. In contrast to CD4^+^CD20^-^ T cells, CD4^+^CD20^+^ T cells were able to induce a fast and strong expression of both adhesion molecules on brain endothelial cells, pinpointing this as a potentially major mechanism of CD4^+^CD20^+^ T cells to advance inflammation in patients with MS and other inflammatory CNS diseases.

Astrocytes are a CNS glial cell that hold a wide range of functions, including formation and maintenance of the BBB. About 97% of astrocytes are in contact with CNS blood vessels where their end feet form the glia limitans, a structure that restricts the entry of peripheral immune cells into the CNS parenchyma after having crossed the endothelial layer. In cooperation with endothelial cells, activated astrocytes can facilitate infiltration of peripheral leukocytes into perivascular spaces and the CNS parenchyma (17, 43). Astrocyte reactivity can be triggered by damage- or pathogen-associated molecular patterns, oxidative stress, or pro-inflammatory cytokines produced by CNS-resident cells or immune cells recruited from the periphery (17, 44). In response to pro-inflammatory TNFα, IFNγ, GM-CSF, and IL-17, astrocytes enhance inflammation by producing additional pro-inflammatory mediators, including chemokines (30, 44). CD4^+^CD20^+^ T cells are known producers of both TNFα, IFNγ, GM-CSF, and IL-17 (21, 45). In our study, culturing astrocytes in CD4^+^CD20^+^ T cell conditioned medium resulted in a substantial production of the chemoattractants CXCL9, CXCL10, CXCL11, and CCL5. CXCL9, CXCL10 and CXCL11 can recruit immune cells expressing CXCR3, which are mainly Th1- and Th17.1-like CD4^+^ T cells and cytotoxic CD8^+^ T cells. These T cell subsets are all central effector cells in the immunopathogenesis of MS. A large proportion of infiltrating CXCR3^+^ T cells in inflammatory tissue as well as in MS lesions co-express the CCL5 receptor CCR5. Furthermore, CXCR3 and CCR5 have previously been shown to cooperate in promoting the homing of T cells to tissues during inflammation (46–48). Therefore, we propose that another mechanism used by CD4^+^CD20^+^ T cells to promote inflammation in the CNS in early disease stages is to induce astrocytes to produce chemokines in order to induce or amplify recruitment of immune cells. Furthermore, a previous study showed that CXCR3^+^ T cells continuously accumulate in MS lesion areas where astrocytes produce CXCL10 (32). Considering a possible role of CD4^+^CD20^+^ T cells in inducing CXCL10-production by astrocytes, this may imply that CD4^+^CD20^+^ T cells are involved in promoting chronic inflammation.

A final discovery is the observation that CD4^+^CD20^+^ T cell-conditioned medium induced HLA-DR mRNA expression in brain endothelial cells, as observed in an RNA-array. This finding was further validated on the protein level by flow cytometry. Expression of the MHC class II molecule HLA-DR on activated brain endothelium cells has previously been shown to enable presentation of myelin antigens, originating from internalized and processed myelin debris, leading to recruitment of myelin-specific CD4^+^ T cells (29). In this way, damage to myelin, as observed in MS, together with activation of CD4^+^CD20^+^ T cells promoting MHCII-expression on endothelial cells, could enhance the CNS antigen-specific T cell response and progression of the nervous tissue damage. We also found that CD4^+^CD20^+^ T cell conditioned medium strongly increased the surface level of MHC class I on the brain endothelial cells. The impact of this observation; however, awaits future clarification.

In conclusion, we propose that CD8^+^CD20^+^ T cells are part of physiological CNS immunosurveillance and may potentially induce the initial steps leading to neuroinflammation in RRMS. Furthermore, our experiments show that activated CD4^+^CD20^+^ T cells can stimulate brain endothelial cells to upregulate adhesion molecules and chemokines needed for peripheral immune cells to adhere and migrate through the BBB, and astrocytes to produce chemokines directing transmigrating cells to the site of inflammation. Also, induction of MHC on endothelial cells may amplify the recruitment and activation of CNS-specific T cells.

## Data Availability

The raw data supporting the conclusions of this article will be made available by the authors, without undue reservation.

## Supplementary material

Supplementary Table 1 Demographic characteristics ^a^ P values are derived from the Mann-Whitney U test. *ns*, non-significant; CSF, cerebrospinal fluid; RRMS, patients with relapsing remitting multiple sclerosis.
