# Supplementary figures and images for: A role of CD20+ T cells in early multiple sclerosis
Source: Front Immunol. 2025 May 19;16:1582535. doi: 10.3389/fimmu.2025.1582535 (PMC12127330; doi:10.3389/fimmu.2025.1582535)

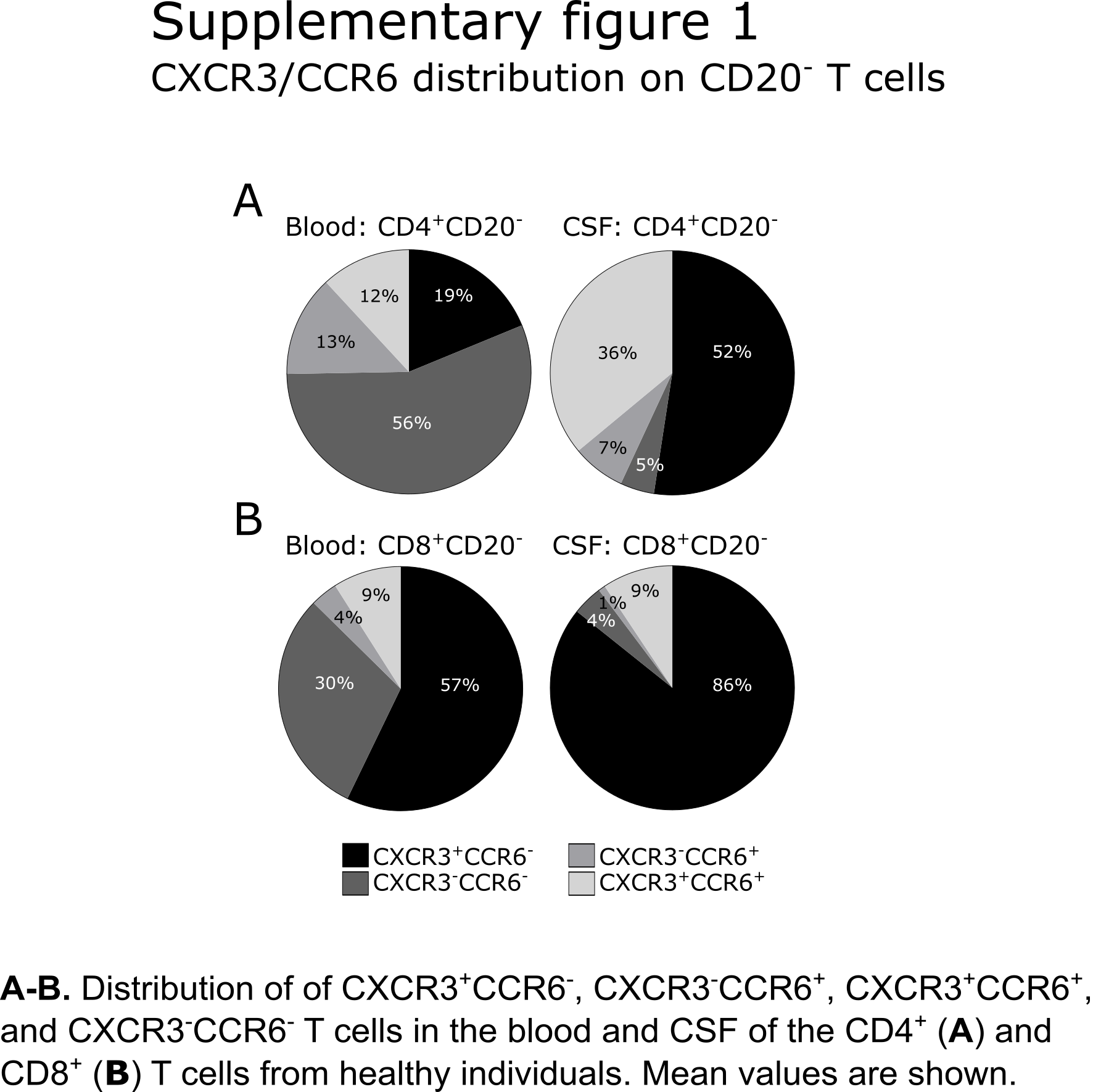

Supplement: Supplementary Table 1 — Demographic characteristics a P values are derived from the Mann-Whitney U test. ns, non-significant; CSF, cerebrospinal fluid; RRMS, patients with relapsing remitting multiple sclerosis. [file Image1.tiff]

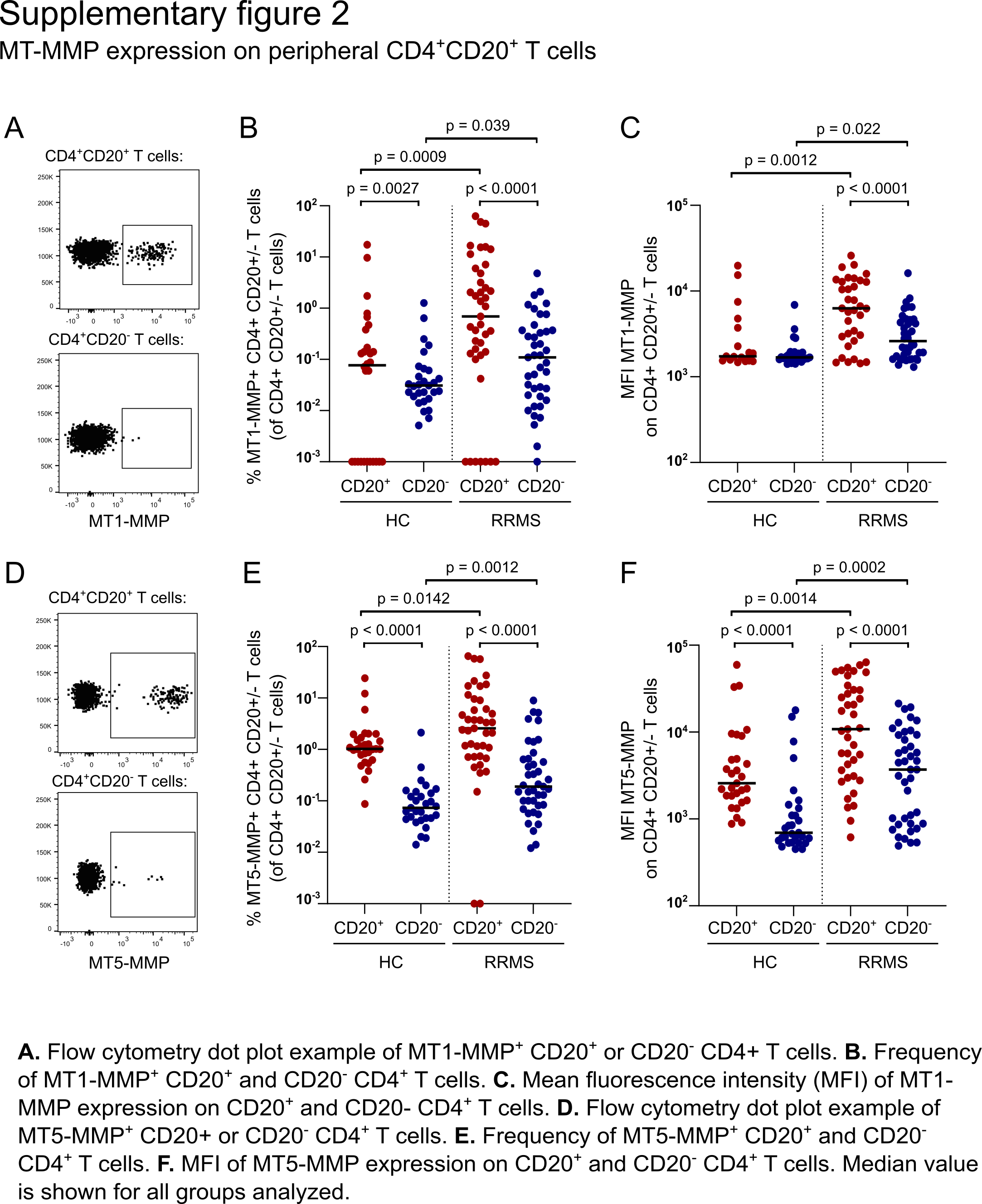

Supplement: Supplementary file 2 [file Image2.tiff]

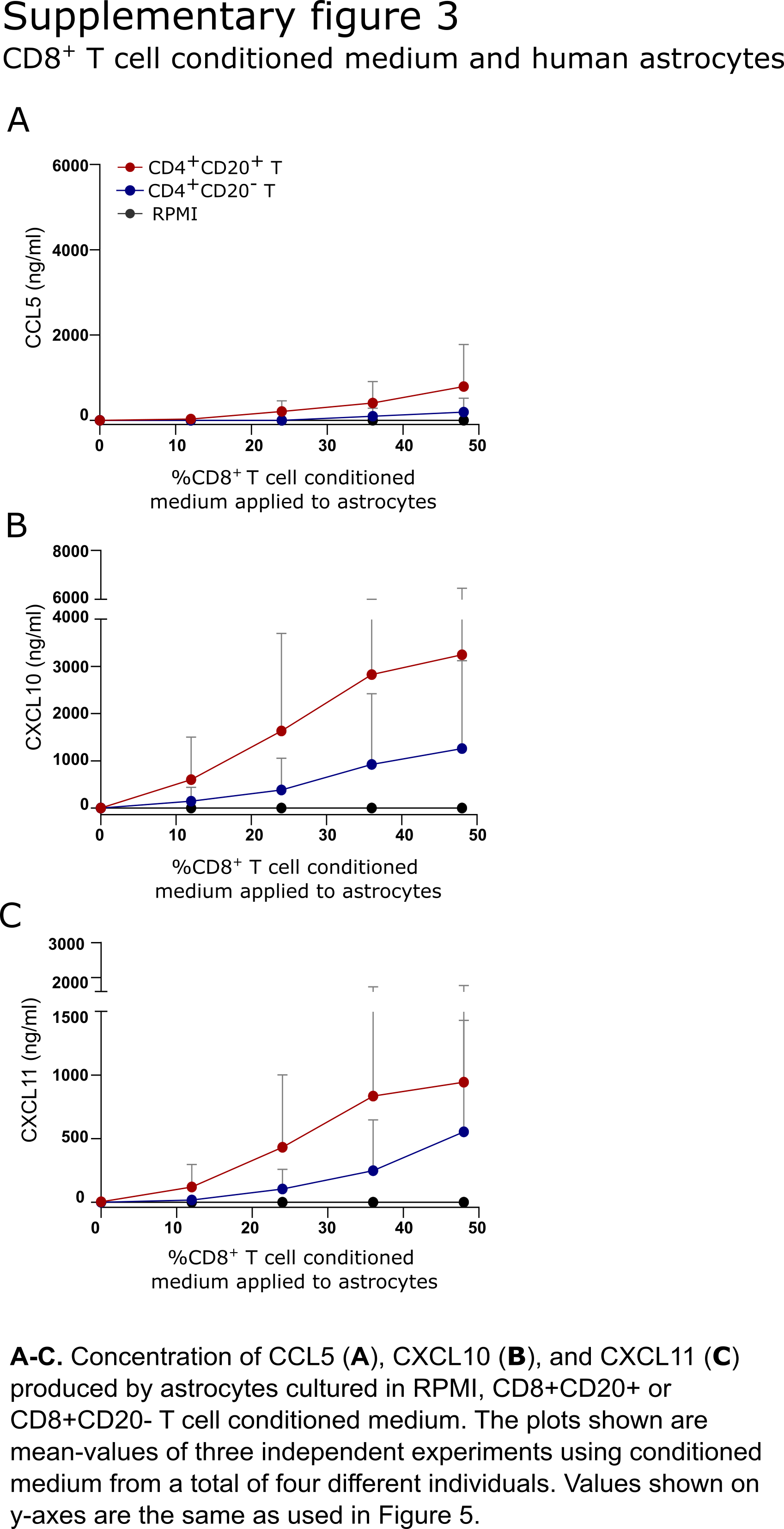

Supplement: Supplementary file 3 [file Image3.tiff]
