# Supplementary material for: A role of CD20+ T cells in early multiple sclerosis
Source: Front Immunol. 2025 May 19;16:1582535. doi: 10.3389/fimmu.2025.1582535 (PMC12127330; doi:10.3389/fimmu.2025.1582535)
Supplement: Supplementary file 4 [file Table1.docx]

**Supplementary Table 1. Demographic characteristics**

|  | n | Age  (mean; Q1-Q3) | | Age  *P* value ^a^ | Sex (female/male) | Sex  *P* value ^a^ | |
| --- | --- | --- | --- | --- | --- | --- | --- |
| **Cohort 1: freshly drawn CSF samples** |  |  | |  |  |  | |
| Healthy individuals | 12 | (49 y; 35-61) | |  | (8/4) |  | |
| **Cohort 2: freshly drawn CSF samples** |  | |  | |  |  |  |
| Symptomatic controls | 10 | (37 y; 27-45) | |  | (3/7) |  | |
| Patients with early RRMS | 16 | (37 y; 31-45) | | *ns* | (11/5) | *ns* | |
| **Cohort 3: freshly drawn blood samples** |  |  | |  |  |  | |
| Healthy individuals | 29 | (36 y; 29-42) | |  | (17/12) |  | |
| Patients with early RRMS | 41 | (36 y; 27-43) | | *ns* | (27/14) | *ns* | |
| **Cohort 3: cryopreserved blood cells** |  |  | |  |  |  | |
| Healthy individuals | 12 | (41 y; 35-44) | |  | (5/7) |  | |
| Patients with RRMS | 12 | (38 y; 30-44) | | *ns* | (9/3) | *ns* | |

^a^ P values are derived from the Mann-Whitney U test. *ns* = non-significant.

Abbreviations: CSF, cerebrospinal fluid; RRMS, patients with relapsing remitting multiple sclerosis.
